# Alterations of oral microbiota distinguish children with autism spectrum disorders from healthy controls

**DOI:** 10.1038/s41598-018-19982-y

**Published:** 2018-01-25

**Authors:** Yanan Qiao, Mingtao Wu, Yanhuizhi Feng, Zhichong Zhou, Lei Chen, Fengshan Chen

**Affiliations:** 1 Department of Orthodontics, School and Hospital of Stomatology, Tongji University, Shanghai Engineering Research Center of Tooth Restoration and Regeneration, Shanghai, 200072 China; 2 Department of Endodontics, School and Hospital of Stomatology, Tongji University, Shanghai Engineering Research Center of Tooth Restoration and Regeneration, Shanghai, 200072 China; 3 Department of Periodontics School and Hospital of Stomatology, Tongji University, Shanghai Engineering Research Center of Tooth Restoration and Regeneration, Shanghai, 200072 China

## Abstract

Altered gut microbiota is associated with autism spectrum disorders (ASD), a group of complex, fast growing but difficult-to-diagnose neurodevelopmental disorders worldwide. However, the role of the oral microbiota in ASD remains unexplored. Via high-throughput sequencing of 111 oral samples in 32 children with ASD and 27 healthy controls, we demonstrated that the salivary and dental microbiota of ASD patients were highly distinct from those of healthy individuals. Lower bacterial diversity was observed in ASD children compared to controls, especially in dental samples. Also, principal coordinate analysis revealed divergences between ASD patients and controls. Moreover, pathogens such as *Haemophilus* in saliva and *Streptococcus* in plaques showed significantly higher abundance in ASD patients, whereas commensals such as *Prevotella*, *Selenomonas*, *Actinomyces*, *Porphyromonas*, and *Fusobacterium* were reduced. Specifically, an overt depletion of Prevotellaceae co-occurrence network in ASD patients was obtained in dental plaques. The distinguishable bacteria were also correlated with clinical indices, reflecting disease severity and the oral health status (*i.e*. dental caries). Finally, diagnostic models based on key microbes were constructed, with 96.3% accuracy in saliva. Taken together, this study characterized the habitat-specific profile of the oral microbiota in ASD patients, which might help develop novel strategies for the diagnosis of ASD.

## Introduction

Autism spectrum disorders (ASD) are complex neurodevelopmental disorders unfolded in the first couple years of life, and characterized by impairments in language and social interactions, often with restricted interests and repetitive behaviors^1^. ASD affect 1-in-68 children in America, with the prevalence increasing dramatically over the past decades^2^. Currently, the fifth edition of Diagnostic and Statistical Manual of Mental Disorders (DSM-5) is considered the “gold standard” for ASD diagnosis. However, DSM-5 encompasses descriptive criteria merely based on empirical data, with no laboratory or other diagnostic tests^3^. As a result, diagnosis according to the relatively time-consuming DSM-5 guideline is not sufficiently scientific, and needs further validation^4^. Thus, developing precise and reliable diagnosis tools for ASD remains a challenge.

Genetic and environmental factors both play roles in the pathogenesis of ASD^5^. Available twin studies showed that environmental factors are more important than genetic predisposition^6^. Among such factors, microbial dysbiosis is of increasing interest, with accumulating reports in animal models and human epidemiologic studies linking disruptive alterations in the gut microbiota to ASD symptomology^7–12^. Although the mechanisms by which gut microorganisms might impact the central nervous system (CNS) are not fully understood, the human microbiota and related metabolites have been reported to affect a variety of complex behaviors, including social, emotional, and anxiety-like behaviors, also contributing to brain development and modulating cognition through imbalances in the microbiota-gut-CNS axis^2,13,14^. Consequently, microbial dysbiosis might play a causal role in the development of mental disorders, in a pathway mediated through the host’s metabolism.

While growing evidence suggests a key role for the gut microbiota in ASD, the neurologic effects of microorganisms inhabiting the oral cavity have been overlooked. Oral microbial communities can undergo dynamic fluctuations in response to various intrinsic and extrinsic factors and these may in turn affect brain function^15^. The oral cavity harbors approximately 700 predominant taxa, and serves as a reservoir for infection at distant body sites, including the neural system^2,16^. Current studies have documented the effects of the oral microbiota on neural function^17–21^. For example, oral microbial dysregulation is associated with Parkinson’s disease, Alzheimer’s disease, multiple sclerosis, and migraine^17–21^. However, the association of oral microbiota shifts with ASD remains unexplored.

Compared to the gut, the oral cavity is anatomically characterized as a unique and complex habitat because of the presence of both soft (i.e. mucosa) and hard (teeth) tissues. Microbiota colonize different oral habitats which are considered different physical niches; these organisms differ markedly in community structure and interact with each other continuously^22^. Additionally, oral samples, especially saliva specimens, are easier to collect compared to stool or intestinal mucosal biopsies. Therefore, it is anticipated that there would be great public interest in oral sampling-based research and clinical diagnosis, particularly for diseases for which sampling otherwise is challenging, such as ASD.

In this study, we collected samples from two distinct intraoral habitats, including saliva and dental plaques, in children with and without ASD. Microbial populations were assessed by 16S rRNA gene sequencing. Dental plaques retain diverse microorganisms and are known to reflect poor dental hygiene and oral health behavior, which were reported to be greatly inadequate in ASD children, consistent with our clinical observation and the current literature^23,24^. On the other hand, saliva, as a representative of the overall oral microbial communities, has been widely used to explore the associations of the oral microbiota with epidemiological characteristics and systemic diseases^25,26^. To our knowledge, this study provides the first evidence for oral microbial alterations in children with ASD, which would help expand the understanding of the relationship between oral microbiota and ASD, and develop more objective strategies for ASD diagnosis.

## Methods

### Ethics Statement

This study was approved by the Ethics Committee of the School and Hospital of Stomatology, Tongji University, and conducted in compliance with relevant guidelines and regulations. Written informed consent was obtained from the parents or guardians of all subjects, prior to the study.

### Subject Recruitment

Children diagnosed with ASD by meeting the DMS-5 criteria were recruited locally from Shanghai Children’s Medical Center. Diagnosis was further confirmed at Shanghai Mental Health Center with the criteria for the International Classification of Diseases and Related Health Problems, Tenth Revision (ICD-10). The enrolled ASD subjects were between 7 and 14 years of age, with no previous medical treatment (except for rehabilitation training) or antibiotic/antifungal use within 3 months of sample collection. Gender- and age-matched healthy children, who were unrelated to the autistic individuals, were recruited from primary schools. The detailed eligibility criteria are presented in Supplementary Table S1. The parents or guardians of the enrolled subjects were asked to fill out the Aberrant Behavior Checklist (ABC) questionnaire to preliminarily evaluate the severity of ASD^27^.

### Sample Collection and Oral Examination

The participants were instructed to refrain from eating and drinking, and to avoid oral hygiene practice, for at least 3 hours prior to sample collection. Before dental sampling, approximately 1 ml of non-stimulated, naturally outflowed saliva was consistently collected and transferred into 1.5 ml sterile Eppendorf tubes (Axygen, CA, USA). Then, the first permanent molars were isolated using cotton rolls and gentle air-drying. Supragingival plaques were obtained separately from caries-free molars in four quadrants (upper right, upper left, lower right and lower left) per subject using sterile Gracey curettes, and pooled in 1.5 ml sterile Eppendorf tubes containing sterile phosphate-buffered saline (PBS). All samples were immediately placed on ice, transported to the local laboratory within 2 hours, and stored at −80 °C until DNA extraction. In total, 111 specimens from 32 ASD children and 27 healthy children were obtained.

The 111 samples collected were divided into four groups according to study populations and sampling habitats: HS, salivary samples from healthy controls (n = 27); HP, dental samples from healthy controls (n = 26); AS, salivary samples from ASD patients (n = 32); AP, dental samples from ASD patients (n = 26).

Oral conditions in each subject, including dentition status, plaque index (PLI), bleeding on probing (BOP), gingival index (GI), sulcus bleeding index (BI), probing depth (PD), and decayed-missing-filled teeth/surfaces (DMFT/DMFS), were recorded after sampling (described in Supplementary Table S2). All examinations were performed by the same experienced dentist according to routine criteria.

### Microbial DNA extraction and PCR amplification

DNA from dental and salivary samples was extracted with the OMEGA-soil DNA Kit (OmegaBio-Tek, USA) according to the manufacturer’s instructions. DNA purity was evaluated based on A260/A280 ratio using a NanoDrop2000 Spectrophotometer (Thermo Fisher Scientific, USA). DNA integrity and size were assessed by 1.0% agarose gel electrophoresis. A negative control containing the buffer only was included during DNA extraction and quantification. DNA samples were frozen at −20 °C until use. The V3-V4 region of the 16S ribosomal RNA gene was amplified using primers 338 F (5′-ACTCCTACGGGAGGCAGCA-3′) and 806 R (5′-GGACTACHVGGGTWTCTAAT-3′) via PCR (95 °C for 2 min, followed by 25 cycles at 95 °C for 30 s, 55 °C for 30 s, and 72 °C for 30 s, and a final extension at 72 °C for 5 min)^28^.

### 16S rRNA gene sequencing

PCR amplicons were separated by agarose gel electrophoresis, purified with the AxyPrep DNA Gel Extraction Kit (Axygen Biosciences, Union City, CA, USA) according to the manufacturer’s instructions, and quantified using QuantiFluor™ -ST (Promega, USA). The purified amplicons were pooled in equimolar and paired-end sequences (2 × 250) on a MiSeq platform (Illumina, USA), according to standard protocols. Raw reads were deposited in the NCBI SRA database (Accession number SRP097646).

### Processing of sequencing data

Raw FASTQ files were demultiplexed and quality-filtered using QIIME (version 1.9.1), with the following criteria: (1) 300 bp reads were truncated at any site receiving an average quality score <20 over a 50 bp sliding window, and truncated reads shorter than 50 bp were discarded; (2) exact barcode matching, with 2 nucleotide mismatch in primer matching and reads containing ambiguous characters removed; (3) only sequences overlapping over more than 10 bp were assembled according to the overlapping sequence. Reads which could not be assembled were discarded. High-quality reads were clustered into operational taxonomic units (OTUs) with the UPARSE software^29^. The taxonomy of each 16S rRNA gene sequence was analyzed by RDP Classifier (http://rdp.cme.msu.edu/) against the Human Oral Microbiota Database, a curated dataset for oral taxa^30^.

### Statistical and Data Analyses

Demographics and clinical parameters of subjects between the ASD and healthy groups were compared by the t-test (age, BMI), Chi-square test (gender) and Mann-Whitney U-test (DMFT/DMFS, PLI, BOP, GI, BI, PD). p < 0.05 was considered statistically significant. Rarefaction curves were generated with Mothur to assess the current sequencing depth. At a 97% level of nucleotide similarity cutoff, the OTUs were used for assessing α-diversity indices (ACE, Shannon and Shannoneven) and Good’s coverage. Phylogenetic α-diversity measures, such as principal coordinate analysis (PCoA), were conducted according to (un)weighted UniFrac distances based on the detected genera in each sample. Independent Student’s t-test and Permutational Multivariate Analysis of Variance (PERMANOVA) were applied to evaluate α and β diversity among different groups. Histograms based on different taxonomic levels were generated to depict the overall microbial composition and abundance. The data of RDP taxonomy were used in LEfSe (http://huttenhower.sph.harvard.edu/lefse/) to identify the taxa differentiating the microbial communities specific to different groups^31^. Microbiota features of ASD subjects were compared to those of healthy controls by the Wilcoxon rank sum test using p-values and FDR Q-values (modified version of FDR^32^) for non-normally distributed variables. Only OTUs with p < 0.05 and FDR Q < 0.05 were considered significant and used in the network analysis. Spearman’s rank correlation coefficients were determined between the OTUs in dental and salivary samples from healthy children and autistic patients. Then, co-occurrence networks were generated using Cytoscape (version 3.4.0). Associations of microbes with oral health indices were assessed by Spearman’s rank correlation, and visualized by heat maps. The Random Forest algorithm and 10-fold cross validation were used to select microbial markers and their combinations. The obtained combinations were assessed by Receiver Operator Characteristic (ROC) analysis for performance^33^. All analyses were performed in the R software package (version 3.3.1; The R Project for Statistical Computing, http://www.R-project.org).

## Results

### Overall structure of the oral microbiota

The demographic and clinical characteristics of all participants are depicted in Table 1 and Supplementary Table S2. To investigate the shifts in structure and composition of oral microbial communities in ASD, 111 samples were sequenced by the Illumina MiSeq technology. After processing, a total of 3,769,401 valid reads were obtained, with an average of 33,959 ± 4,253 reads per sample. All sequences (excluding singletons) were classified into 405 OTUs at a 97% similarity level, representing 12 phyla, 132 genera and 308 species. The predominant taxa were similar to those previously reported in non-ASD populations^25,34^, and are shown in Supplementary Fig. S2. The Good’s coverage was 99.86%, and rarefaction curves approached asymptotes for most samples with the current sequencing (Supplementary Fig. S1).

**Table 1:** Demographics and clinical parameters of the subjects.

Characteristics	Salivary samples				Dental samples	
	ASD patients(n = 32)	Healthy control(n = 27)	p value	ASD patients(n = 26)	Healthy control(n = 26)	p value
Age (Mean ± SD)	10.02 ± 1.43	10.19 ± 0.59	0.55	10.15 ± 1.35	10.37 ± 0.66	0.46
Female/Male	5/27	6/21	0.52	4/22	2/22	0.74
Body mass index (Mean ± SD)	19.59 ± 3.60	19.92 ± 4.53	0.77	19.98 ± 3.85	20.49 ± 3.43	0.63
Disease severity score (Mean ± SD)	78.66 ± 26.46	N/A		78.15 ± 24.95	N/A	
Decayed-missing-filled teeth (Mean ± SD)	2.03 ± 1.79	1.04 ± 1.86	<0.01	2.27 ± 1.74	0.96 ± 1.79	<0.01
Decayed-missing-filled surfaces (Mean ± SD)	3.91 ± 3.83	1.96 ± 3.86	<0.01	4.42 ± 3.85	1.81 ± 3.72	<0.01
Plaque index (Mean ± SD)	1.88 ± 0.43	1.88 ± 0.34	0.88	1.98 ± 0.35	1.91 ± 0.42	0.8
Gingival index (Mean ± SD)	0.90 ± 0.44	0.25 ± 0.47	<0.01	0.96 ± 0.44	0.29 ± 0.47	<0.01
Sulcus bleeding index (Mean ± SD)	1.02 ± 0.72	0.25 ± 0.47	<0.01	1.11 ± 0.75	0.29 ± 0.47	<0.01
Bleeding on probing (+/−)	0.69 ± 0.56	0.31 ± 0.45	0.01	0.75 ± 0.56	0.33 ± 0.44	<0.01
Probing depth/mm (Mean ± SD)	1.92 ± 0.37	1.81 ± 0.47	0.53	1.92 ± 0.36	1.78 ± 0.44	0.47

**Figure 1:**
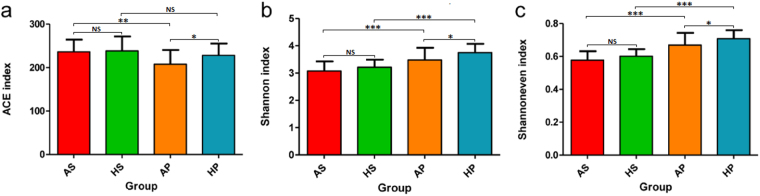
Overall structural comparison of the oral microbiota. Sequences were randomly subsampled to obtain equal numbers of sequences (21,795) from each dataset. ACE index (**a**, representing community richness), Shannon index (**b**, representing diversity), and Shannoneven index (**c**, representing evenness) were calculated for dental and salivary samples. The bars depict mean ± SD of relative abundance rates. *p < 0.05; **p < 0.01; ***p < 0.001; NS, p > 0.05. HS (saliva samples collected from healthy controls, n = 27); HP (plaque samples from healthy controls, n = 26); AS (saliva samples from ASD patients, n = 32); AP (plaque samples collected from ASD patients, n = 26).

**Figure 2:**
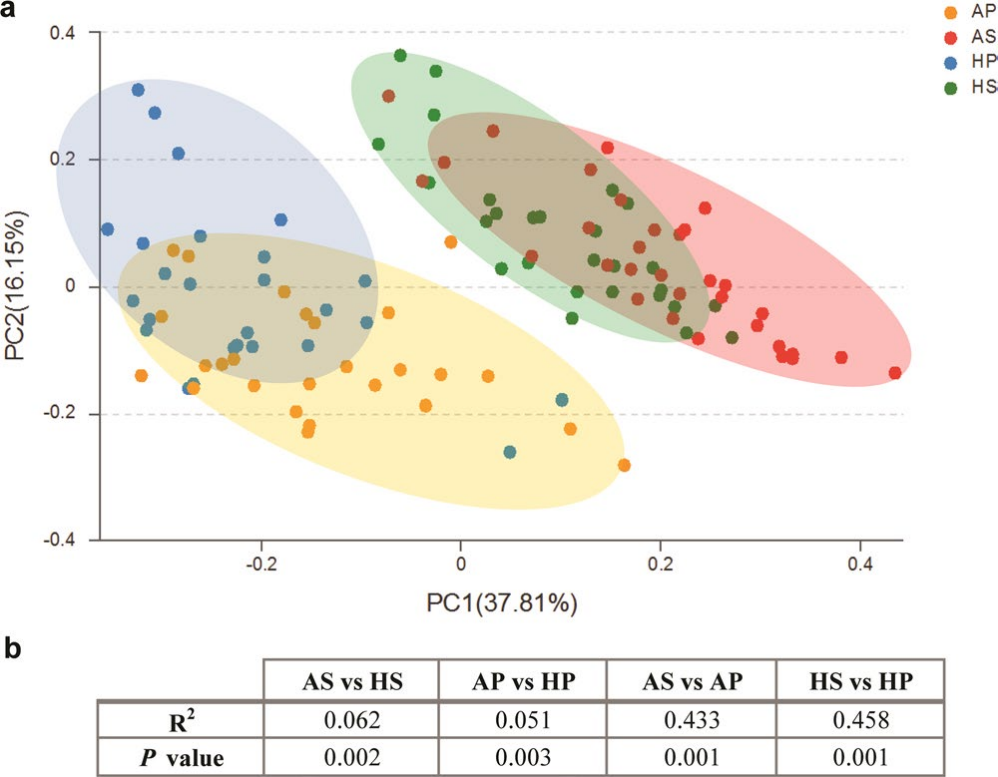
Comparison of β-diversity of the oral microbiota. A principal coordinate analysis (PCoA) score plot for all samples (principal coordinates 1 and 2) based on weighted UniFrac distance was generated to depict the divergence in distribution of the oral microbiota (**a**). Each point represents the oral microbiota composition of one sample. PERMANOVA shows statistically significant community differences between ASD and healthy controls within each habitat, as well as between saliva and dental plaques, regardless of the disease status (**b**).

Alpha diversity (Fig. 1a–c and Supplementary Table S3), richness index (ACE), diversity index (Shannon), and evenness index (Shannoneven) were all significantly lower in ASD subjects compared to healthy controls (P < 0.05) in dental samples; however, in saliva samples, none of these indices showed significant differences (P > 0.05), although a downward trend was observed. Dental plaques displayed a higher level of bacterial diversity than saliva, with a lower level of bacterial richness (Fig. 1a,b). Moreover, principal coordinate analysis (PCoA) based on (un)weighted UniFrac metrics was performed to identify any differences in organismal structure of oral microbiota (Fig. 2a, Supplementary Fig. S3). The salivary and dental bacterial communities showed non-overlapping clustering, revealing that the oral microbiota mainly differed by habitats rather than the disease status.

We also observed divergences in bacterial communities between ASD patients and healthy subjects in both salivary and dental samples, with specimens from healthy subjects tending to cluster more closely (Fig. 2a). The PERMANOVA identified significant differences in PCoA plots between ASD and healthy children in both habitats (P < 0.01; Fig. 2b).

### Taxonomy-based analysis of microbial changes in ASD patients

**Figure 3:**
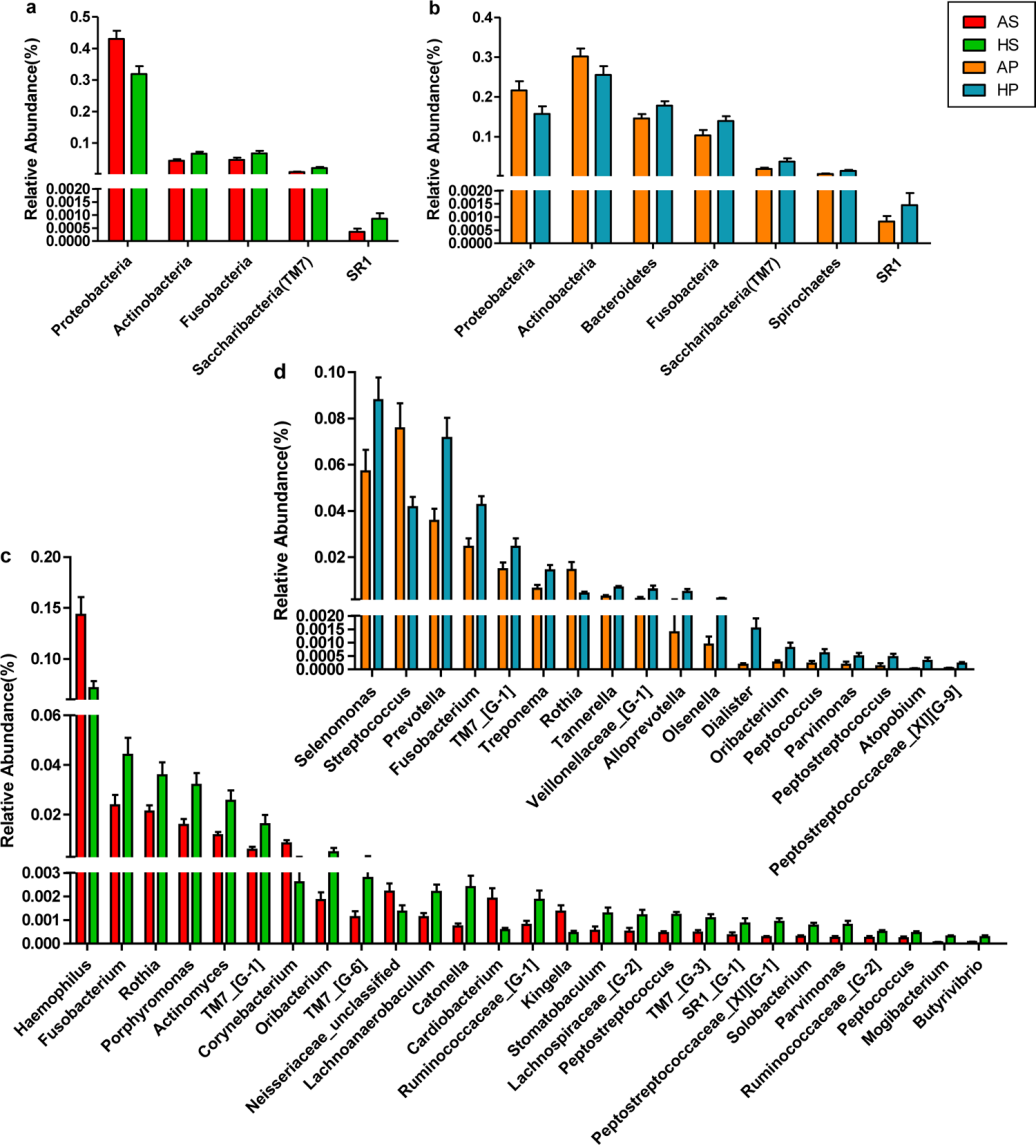
Changes of oral microbiota at the phylum and genus levels between ASD and control subjects. The graphs depict Wilcoxon rank sum test results with p < 0.05 and FDR Q < 0.05 from comparisons of relative abundance between ASD and healthy children at the bacterial phylum level in saliva (**a**) and plaque (**b**) samples. Similar comparisons at the genus level in saliva and plaque samples are shown in (**c**) and (**d**), respectively (p < 0.05 and FDR Q < 0.05).

To identify the distinguishing taxa of saliva and dental plaque samples, LEfSe was performed. The cladogram based on LDA score >2 showed multiple discriminative features of saliva and the dental microbiota between healthy and ASD subjects at different taxonomic levels (Supplementary Fig. S4). More distinguishable taxa between ASD and control groups were observed in saliva compared to dental samples. Then, taxa with significant differences were further assessed by the Wilcoxon rank-sum test, with stringent criteria (P < 0.05 and FDR Q < 0.05). Despite high inter-individual variability, predominant phyla in all groups were Firmicutes, Proteobacteria, Actinobacteria, Bacteroidetes and Fusobacteria, constituting 98.06% and 94.86% of salivary and dental microbes, respectively. The phylum Proteobacteria was more abundant in ASD patients (both in salivary and dental samples) compared to controls (FDR Q = 0.017 and 0.014, respectively), while the other phyla were more abundant in healthy controls (Fig. 3a,b). Meanwhile, some genera differed significantly in abundance between ASD and healthy children (Fig. 3c,d). Specifically, among genera with a mean relative abundance >1%, ASD was associated with increased abundance rates of *Streptococcus* (FDR Q = 0.02 in plaques) and *Haemophilus* (FDR Q = 0.007 in saliva), and decreased rates of *Prevotella* (FDR Q = 0.009 in plaques), *Selenomonas* (FDR Q = 0.042 in plaques), *Actinomyces* (FDR Q = 0.002 in saliva), *Porphyromonas* (FDR Q = 0.03 in saliva), and *Fusobacterium* (FDR Q = 0.011 and 0.025 in plaques and saliva, respectively). Interestingly, in children with ASD, *Rothia* showed a significantly higher abundance in plaques (FDR Q = 0.023) but lower abundance in saliva (FDR Q = 0.033) compared to controls, indicating that this genus might play habitat-specific roles in ASD children.

### Co-occurrence networks of salivary and dental bacterial communities in patients with ASD and healthy controls

**Figure 4:**
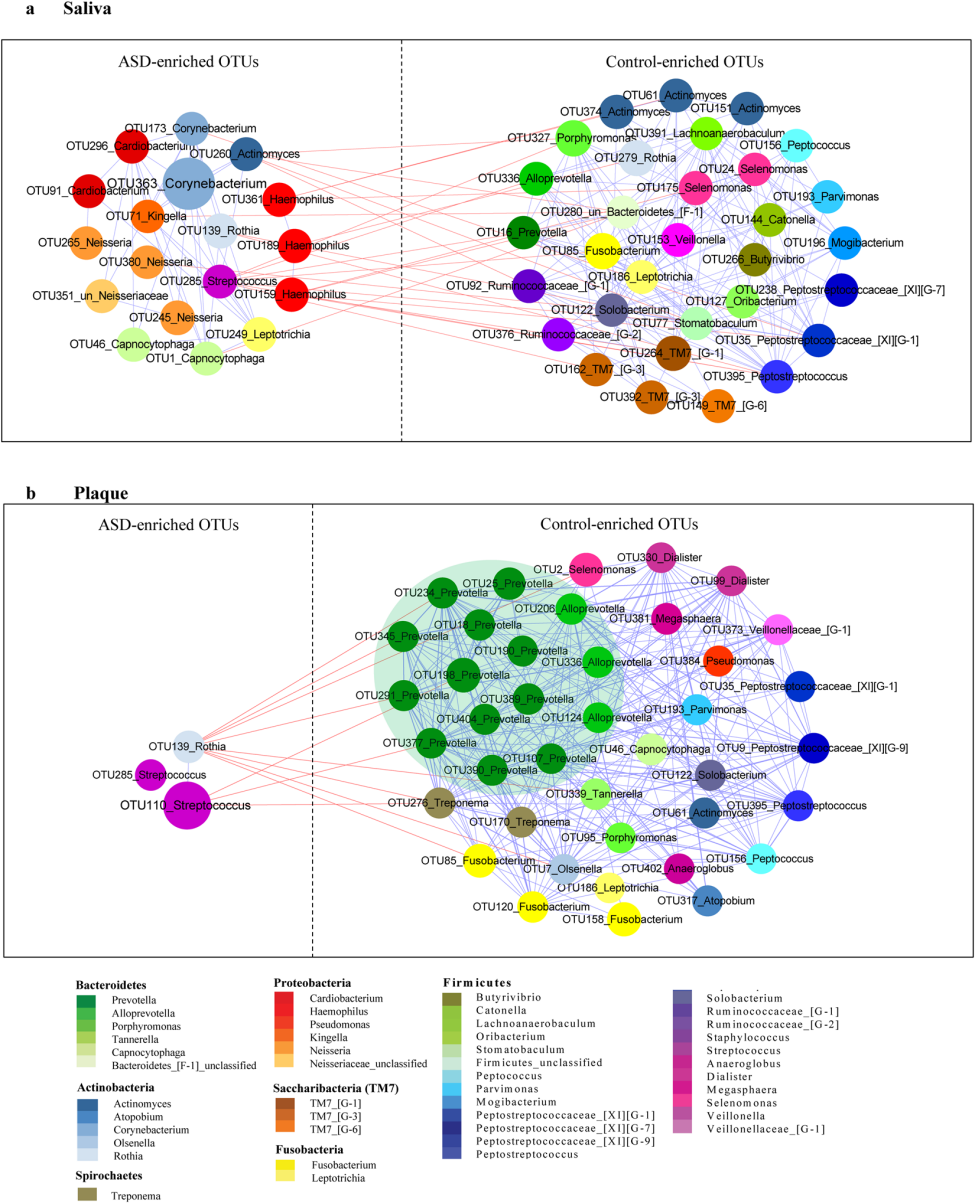
OTUs enriched in salivary and dental samples in ASD patients and healthy controls. A co-occurrence network was deduced from 53 OTUs in salivary samples (**a**) and 43OTUs in dental samples (**b**). Nodes depict OTUs with IDs and affiliated genus names displayed in the center. The size of the nodes indicates the abundance of each OTU. The color of a node indicates its taxonomic assignment. Connecting lines represent Spearman correlation coefficients above 0.5 (blue) or below −0.5 (red).

The differential OTUs identified by the Wilcoxon rank-sum test (P < 0.05, FDR Q < 0.05, Supplementary Tables S4, S5) in salivary and dental samples (53 and 43 OTUs, respectively) were used for network analysis. By assessing Spearman’s correlation coefficients for all OTU markers, a network plot displaying potential co-occurrence relationships was generated (Fig. 4, Spearman’s correlation coefficients |r| >0.5). OTUs were more highly interconnected in control groups than in ASD counterparts, especially in dental samples, indicating that ASD was not only associated with decreased richness of commensals, but also related to reduced mutual effects within these bacteria. Among these commensals, an interesting finding was the obvious reduction of Prevotellaceae co-occurrence network patterns in dental samples, including *Prevotella* and *Alloprevotella* clusters (Fig. 4b). In contrast, the OTUs in ASD groups formed smaller clusters and were less interconnected. Two ASD-enriched shared OTUs, including OTU139 (*Rothia aeria*) and OTU285 (*Streptococcus mutans*), and saliva-specific OTUs, *i.e*. OTU159, OTU189, and OTU361, which were all annotated to *Haemophilus*, were negatively correlated with control-enriched OTUs, suggesting unknown antagonistic or mutually exclusive relationships. Moreover, clusters containing 9 OTUs belonging to *Actinomyces*, *Solobacterium*, *Alloprevotella*, *Leptotrichia*, *Peptostreptococcus* and *Peptostreptococcacea [XI][G-1]*, were all enriched in both salivary and dental samples from healthy controls but not in the ASD groups.

**Figure 5:**
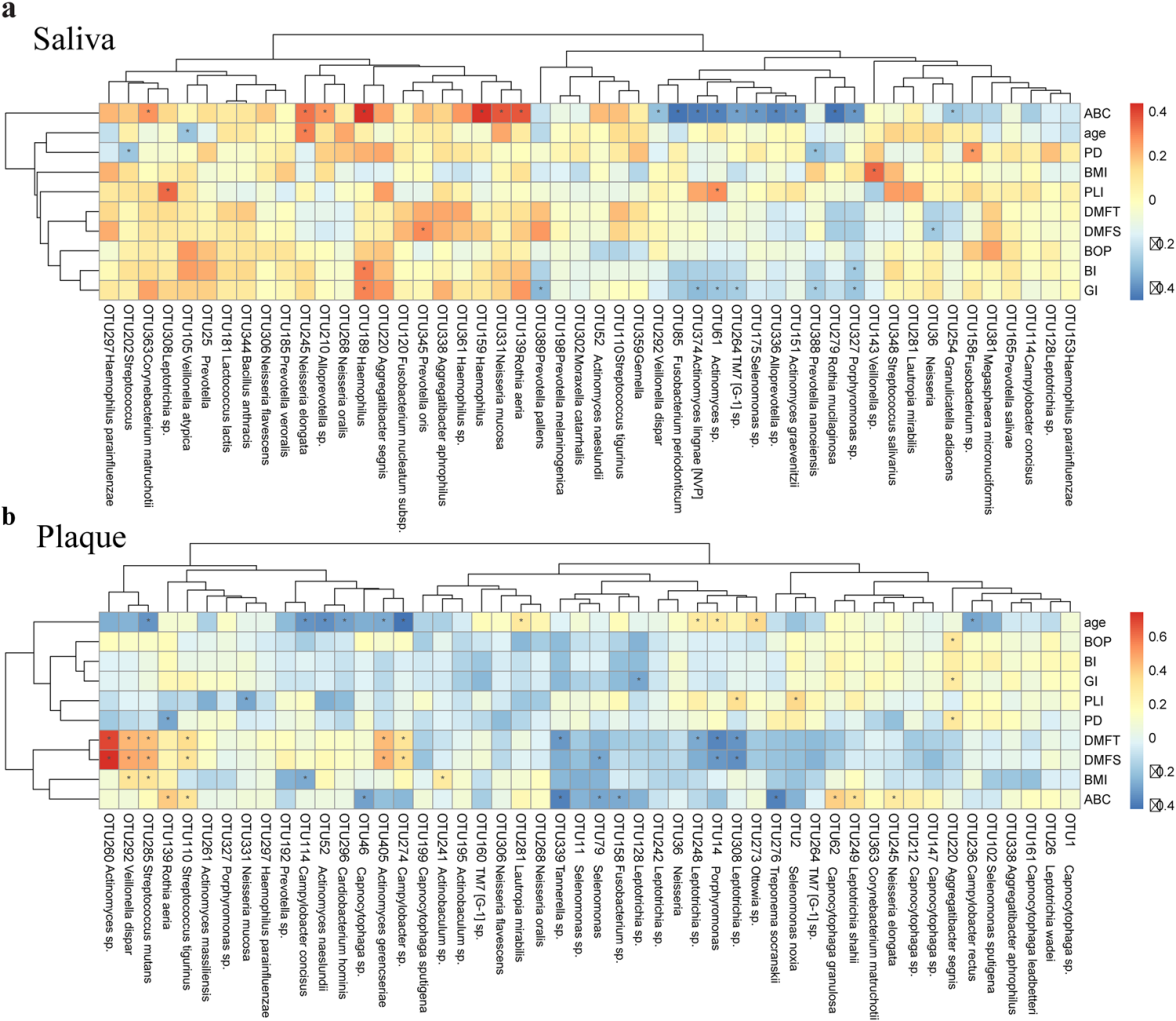
Correlation between OTUs and various clinical indices. The abundance of OTUs (top 50) enriched in saliva (**a**) and dental plaques (**b**) of ASD children and controls were analyzed for covariation with clinical variables using Spearman’s rank correlation. The correlation effect is indicated by a color gradient from blue (negative correlation) to red (positive correlation). Correlation coefficients and p-values (*p < 0.05) are shown. OTUs and clinical variables in the heat maps were ordered using unsupervised hierarchical clustering. BMI, Body Mass Index; DMFT, Decayed-Missing-Filled Teeth; DMFS, Decayed-Missing-Filled Surface; PLI, Plaque Index; GI, Gingival Index; BI, Bleeding Index; PD, Probing Depth; BOP, Bleeding on Probing; ABC score, scale of ASD severity.

Next, co-variations were computed between the relative abundance rates of the most abundant 50 OTUs and clinical indices of ASD as well as oral health-related indices (Fig. 5a,b). A separation was found between control- and ASD-enriched OTUs based on ABC scores, which are used to preliminarily determine the severity of ASD^27^. Notably, most ASD-enriched phylotypes, such as *Haemophilus sp*. (OTU159, R = 0.445, p < 0.001) and *Rothia aeria* (OTU139, R = 0.381, P = 0.004), were positively correlated to the ABC score, to which control-enriched phylotypes were negatively correlated, both in salivary and dental samples. No saliva phylotypes were highly correlated with DMFT/S, which reflects the severity of dental caries. However, in dental plaques, 6 phylotypes belonging to the genera *Streptococcus*, *Actinomyces* and *Capnocytophaga* were positively correlated with DMFT/S. These findings indicated that dental caries had a greater impact on the microbial structure of dental plaques compared to saliva. Furthermore, *Aggregatibacter segnis* (OTU220) was positively correlated to BOP, GI, and PD, which reflects gingival inflammation.

### Diagnostic performance of salivary and dental microbiota

**Figure 6:**
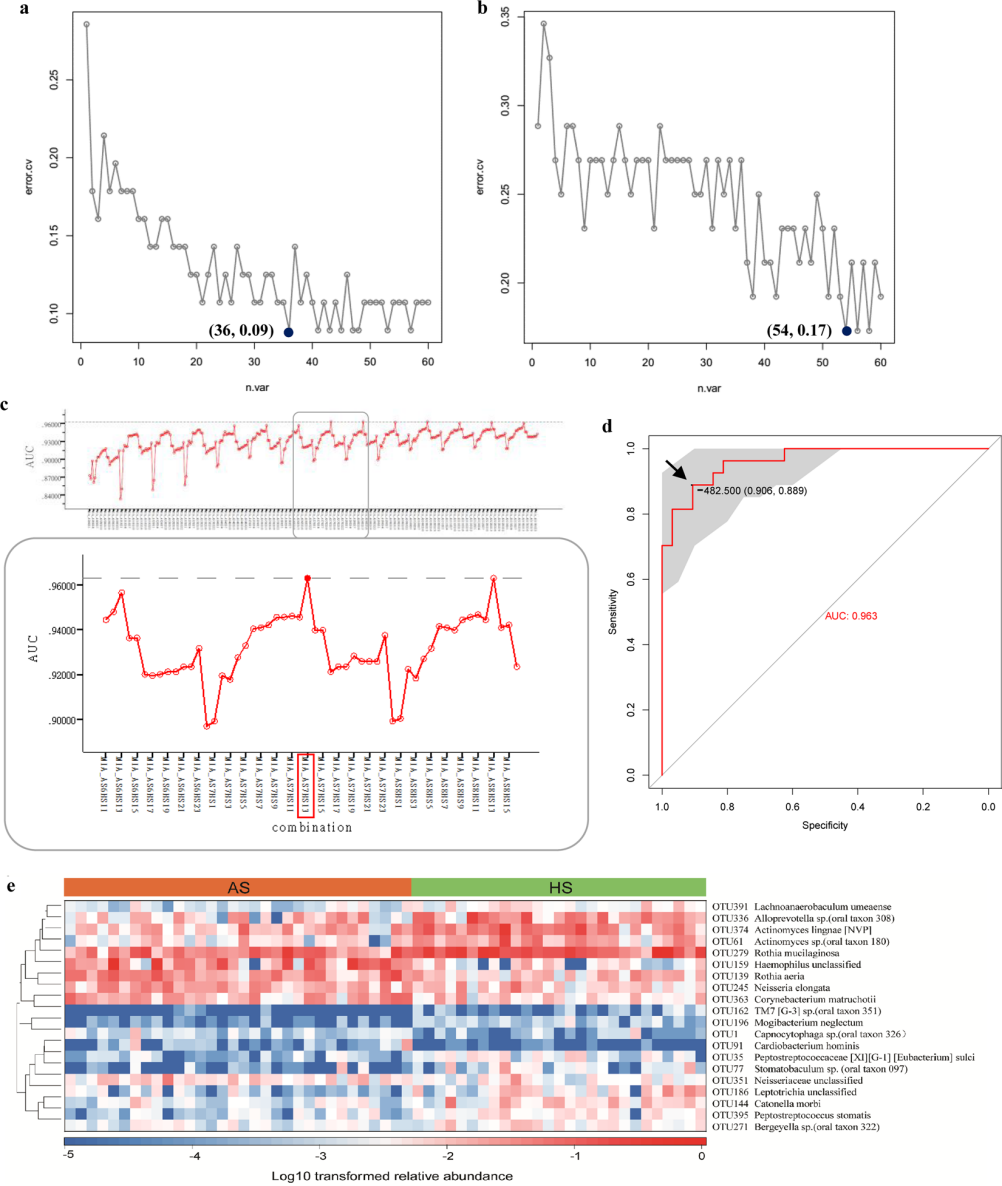
Diagnostic value assessment of salivary and dental samples. Cross-validation error of the random forest ASD classifier was performed based on OTUs in saliva (**a**) and dental plaques (**b**). The grey curve represents a trial of 10-fold cross-validation. The black dot indicates the final number of OTUs used for classification and related errors. (**c**) The AUCs of all combinations in saliva are depicted in the above panel. The following panel shows the magnified portion in the grey rectangle, indicating the AUC of the MIA_AS7HS13 combination reached the highest value. (**d**) The ROC curve of the above MIA in ASD diagnosis is depicted. The area under the ROC curve (AUC) was calculated and is shown in the center. The arrow shows the optimal cut-off point in the salivary microbiota. (**e**) The heat map indicates the ability of salivary potential biomarkers to discriminate between healthy and ASD subjects.

We subsequently performed the Random Forests (RF) analysis to examine whether ASD and healthy children could be discriminated based on microbiota composition. RF analysis assigned a variable importance score to each OTU, according to the extent of its contribution to the model. Based on the variable importance profile, we selected sixty OTUs with the highest variable importance sores for tenfold cross-validation, which was used to define the minimum number of top-ranking taxa according to decrease in classification error rate. Finally, 36 OTUs were identified in saliva with an error of 0.09 and 54 OTUs in dental plaques with an error of 0.17 (Fig. 6a,b). These findings indicated a more accurate discrimination value for salivary microbiota.

Then, we used the top OTUs to construct a prediction model for the diagnosis of ASD. To facilitate the clinical application of the identified markers, a more straightforward index, “microbial index of ASD (MIA)”, was developed for the discrimination of patients based on the relative abundance of the above OTUs:

$$MIA=\frac{{\sum }_{i=m}\,{\rm{abundance}}(\text{ASD}\,\text{enriched})\,i}{m}-\frac{{\sum }_{i=n}\,{\rm{abundance}}(\text{control}\,\text{enriched})\,i}{n},$$

After computing all combinations of ASD and control enriched bacteria in saliva and dental plaques, respectively, MIA was used to generate receiver operating characteristic (ROC) curves. The detailed AUC values for all combinations are shown in Supplementary Table S6. In total, 20 key OTUs (7 in ASD patients and 13 in healthy children, Fig. 6c) were obtained in saliva as well as 6 (all in healthy controls, Supplementary Fig. S5) in dental plaques, with areas under the ROC curve (AUCs) reaching the highest value. AUC, cut-off point, sensitivity, specificity and p-values are depicted in Fig. 6d and Supplementary Fig. S5. The area under the ROC curve of MIA in saliva reached 96.3%, and was higher than that of MIA in dental plaques, indicating that the salivary microbiota was more efficient for ASD diagnosis than the dental counterpart. In saliva, the cut-off point for MIA was determined at -482.5 (sensitivity of 0.889 and specificity of 0.906), indicating that individuals with MIA exceeding −482.5 might have a risk of ASD. Moreover, correlations between salivary biomarkers and ASD subjects were obtained in the heat map, indicating the ability of these biomarkers to distinguish healthy from autistic hosts (Fig. 6e).

## Discussion

In this cross-sectional study, an initial comparative analysis of oral microbiota in ASD children with that of age, sex and BMI matched healthy controls was conducted via 16S rRNA gene sequencing. We identified alterations in the composition and taxonomy of the oral microbiota in ASD patients. Previous studies have reported strong correlations between ASD and altered gut microbiota using different methods^7–12,35^. Some of our findings broadly corroborated these reports evaluating intestinal microbiota. For example, several studies assessing the gut microbiota in stool or intestinal biopsies found no visible changes in diversity and richness between ASD subjects and controls (unrelated healthy controls or neurotypical siblings)^7,9,36^; nevertheless, Kang *et al*. reported significantly reduced diversity of gut microbiota in autistic children compared to neurotypical subjects^12^. In this research, our data on richness and diversity showed no differences in salivary samples; however, a minor but statistically significant decrease in dental samples was observed in ASD children, compared to non-ASD controls. Lower bacterial diversity may reduce the microbial integrity and fail to provide resistance to environmental stressors, such as the intake of pathogenic microbes^37^.

In addition, the increasing or decreasing trends of many taxa at different levels in this study were similar to those obtained for intestinal studies of ASD population. Several gut microbiota studies have revealed changes in relative abundance of phylotypes such as Proteobacteria, Bacteroidetes, and Actinobacteria^7,12,38,39^. Consistent with the latter reports, in this research, significantly higher abundance of Proteobacteria (in both salivary and dental samples) and lower abundance rates of Actinobacteria (in saliva) and Bacteroidetes (in dental plaques) were observed in ASD children. At the genus level, increased abundance of *Streptococcus* and *Haemophilus*, as well as markedly reduced abundance of *Prevotella*, *Alloprevotella*, *Selenomonas*, *Actinomyces*, *Porphyromonas* and *Fusobacterium* in ASD samples were observed, suggesting a characteristic dysbiotic signature. The family Prevotellaceae, including the genera *Prevotella* and *Alloprevotella*, also showed a relatively low abundance in children with ASD. This was evident in dental samples as demonstrated in the co-occurrence network (Fig. 4). In agreement with these findings, Finegold *et al*. recorded a depletion of *Prevotella* in the gut of autistic patients compared to sibling controls^38^. Meanwhile, Kang *et al*. reported that *Prevotella*, as the most significantly altered genus between autistic and neurotypical subjects, decreases dramatically in abundance in stool samples from autistic patients^12^. A low abundance of *Prevotella* was also detected in feces from patients with Parkinson’s disease and untreated Multiple Sclerosis, supporting the relevance of this bacterium in CNS disorders^40,41^. *Prevotella* is a commensal microorganism in multiple human habitats, including the intestine and oral cavity; it does not only interact with the immune system but also plays a key role in degrading a broad spectrum of saccharides^42–44^. Interestingly, it was reported that autistic children may have deficiencies in saccharide metabolism and impaired carbohydrate digestion^36,45^. *Prevotella* species also have essential genes for the biosynthesis of vitamins^44^, which were reported to mitigate ASD symptoms^46,47^. Future studies are warranted to further interrogate the role of Prevotellaceae, also evaluating its therapeutic potential for ASD.

In addition to a reduction in autochthonous bacteria, increased amounts of potential pathogens, including *Haemophilus*, *Corynebacterium*, *Cardiobacterium*, *Kingella*, *Streptococcus* and *Rothia*, were observed in ASD patients. These pathogens are quite different from intestinal bacterial pathogens, such as *Clostridium*, *Sutterella*, and *Desulfovibrio*^12,38,48,49^, which were not detected in the present study. In the genus *Haemophilus*, for example, all seven members were more abundant in salivary samples from ASD patients, with OTU159, OTU189 and OTU361 significantly discriminative. They were also positively correlated to ABC scores (Fig. 5a), indicating an association with ASD symptom severity. Their nearest neighbor, *Haemophilus parainfluenzae*, a Gram-negative and facultative anaerobic bacteria, is not only prevalent in oral diseases^50^, but also considered a potential pathogen causing diseases in remote target organs and systems, e.g. bacteremia and infectious endocarditis^51,52^. These evidences indicate that *H. parainfluenzae* and its metabolites could gain access to the bloodstream and trigger disease outbreaks. *H. parainfluenzae* was also reported to bypass the blood-brain barrier (BBB), enter the cerebrospinal fluid, and cause meningitis^53,54^. Thus, we hypothesized that *H. parainfluenzae* and its metabolites in the oral cavity might enter the blood circulation and even cross the BBB to affect brain development in ASD children.

Another putative pathogen showing overgrowth in dental samples was *Streptococcus*, a potent immunogenic trigger that was reported to affect the risk of CNS dysfunction such as Tourette Syndrome (another neurodevelopmental illness), Sydenham chorea and bacterial meningitis^55,56^. Indeed, *Streptococcus* was recorded to cause neurological damage by producing neurotoxins such as streptomycin, streptodornase, and streptokinase^57^. Studies of the intestinal microbiota indicated that *Streptococcus* might be responsible for bacterial infection in Parkinson’s disease and liver cirrhosis, probably originating from the mouth^57–59^. This finding indicated that specific oral bacteria could invade the gut and subsequently influence remote organs. Moreover, *Rothia* was highly abundant in ASD-associated dental samples, but interestingly, showed low abundance in saliva (Fig. 3c,d). This discrepancy could be explained at the species level. Among *Rothia* species, *R. mucilaginosa* (OTU279) abundance was significantly reduced in ASD salivary samples (Supplementary Table S4). However, *R. aeria* (OTU139) was significantly more abundant in ASD children in both habitats and positively correlated to ABC scores (Fig. 5a,b). Several studies have reported *R. aeria* infection in sepsis, arthritis and pneumonia, most of which were associated with dental pathology^60–62^. We conjecture that *Rothia aeria* might be involved in systemic inflammation and immune response in the pathogenesis of ASD.

Based on the putative pathogens identified in this study, we ventured to hypothesize the plausible mechanisms underlying the oral microbial disturbance in the pathogenesis of ASD. It was documented that oral bacteria could easily and frequently gain access to the bloodstream through routine dental procedures such as chewing, brushing and dental treatment, causing bacteremia subsequently^63^. A previous study demonstrated that the most likely pathway for the dissemination of oral microorganisms to the brain is hematogenous spread^64^. The pro-inflammatory responses provoked by oral bacteria might impair BBB integrity, allowing bacteria to reach the brain and quietly contribute to the pathogenesis of neurological diseases^18^. In addition, previous studies of Alzheimer’s disease described neuronal pathways^18,65^. The olfactory and trigeminal nerves may serve as direct passages for oral bacteria to the CNS or as transmitters of neural signals from the oral cavity to the brain. The orofacial region, which is enriched with vessels and nerves, is anatomically close to the brain, facilitating the cross-talk between the oral microbiota and the CNS. However, due to the exploratory nature of this study, the above attempts to explain the mechanisms underlying the link between ASD and the oral microbiota are highly speculative. Further research is needed for better understanding.

ASD diagnosis in clinical practice is currently guided by DSM-5 criteria, which are based on a consensus regarding clusters of clinical symptoms, with no trusted or objective laboratory measure^3^. Its precision and reliability were overstated in the past decades. Therefore, DSM-5 validity has been challenged by several groups, including the National Institute of Mental Health^4^. Although tools have been developed for autism diagnosis, the desire for clinically useful and reliable biomarkers remains strong^66^. In the present study, the MIA diagnosis model based on oral microbial biomarkers was proposed and achieved 96.3% accuracy, showing a great potential for improved diagnostic sensitivity and specificity. Compared to the gut microbiota usually collected from feces or biopsies, the oral microbiota is easier to sample and potentially mirrors the ASD-associated microbiota profile *in situ*. In addition to microbial markers, clinical indices could be combined to further improve the diagnosis of ASD. Among the demographical and clinical variables assessed in this study, age, BMI, PLI and PD showed no significant differences between ASD and control subjects. However, other parameters, including ABC scores (reflecting ASD severity roughly), DMFT/S (reflecting dental caries severity), BI, GI, and BOP (reflecting dental hygiene), differed significantly between the ASD and healthy cohorts, with higher severity in ASD individuals. ASD children were reported to have a relatively poor oral health condition due to inadequate tooth brushing, consistent with our clinical observation^23,24^. This might be related to lack of the necessary manual dexterity of autistic children as well as difficulties encountered by trainers and parents while brushing the children’s teeth. These oral health indicators might be used to evaluate the risk of ASD. Furthermore, inflammatory factors and metabolites in the oral cavity, such as interleukins, lipopolysaccharides and short-chain fatty acids, could also be included in the algorithm for ASD diagnosis to achieve a higher accuracy and more precise results. Future studies are still warranted to select the optimal combination of the indicators discussed above.

In conclusion, we preliminarily identified the oral microbial taxa associated with ASD, and delineated different features of salivary and dental microbiota between ASD and healthy individuals, providing a comprehensive cross-sectional description of oral microbiota alteration in ASD. This study deciphered dysbiosis in the oral microbial community of ASD patients, suggesting a potential role for microorganisms in the progression of this disease, although a causal relationship cannot be discerned from the association alone. We also suggest a diagnostic approach based on oral microbial features, providing new insights into the design of novel and readily accessible strategies for the diagnosis of ASD. Although this was more sensitive and objective compared to the empirical DSM method, verification of the current findings in large cohorts is required.

### Accession codes

The data are available at the NCBI Sequence Read Archive (SRA) with the accession number SRP097646.

## Electronic supplementary material

Dataset 1

Dataset 2
