# Supplementary material for: Alterations of oral microbiota distinguish children with autism spectrum disorders from healthy controls
Source: Sci Rep. 2018 Jan 25;8:1597. doi: 10.1038/s41598-018-19982-y (PMC5785483; doi:10.1038/s41598-018-19982-y)
Supplement: Supplementary file 1 — Dataset 1 [file 41598_2018_19982_MOESM1_ESM.doc]

**Supplementary Information**

**Alterations of oral microbiota distinguish children with autism spectrum disorders from healthy controls**

**Yanan Qiao****^1^, MingtaoWu^2^, Yanhuizhi Feng^3^, Zhichong Zhou^1^, Lei Chen^1^&Fengshan Chen^1^**

^1^Department of Orthodontics, School and Hospital of Stomatology, Tongji University, Shanghai Engineering Research Center of Tooth Restoration and Regeneration, Shanghai, 200072, China. ^2^Department of Endodontics, School and Hospital of Stomatology, Tongji University, Shanghai Engineering Research Center of Tooth Restoration and Regeneration, Shanghai, 200072, China. ^3^Department of Periodontics, School and Hospital of Stomatology, Tongji University, Shanghai Engineering Research Center of Tooth Restoration and Regeneration, Shanghai, 200072, China. Correspondence and requests for materials should be addressed to F.C. (email: [studentboss2017@163.com](mailto:studentboss2017@163.com))

**Supplementary Figures**

**Figure S1** Rarefaction curves of 111 samples. Most curves become flat in the end, indicating that a reasonable number of tags were analyzed.

**Figure S2** Taxon plots depicting community composition of each sample at the phylum (**a**) and genus (**b**) level. The predominant 8 phyla and 50 genera are shown. Each phylotype is indicated by a different color.

**Figure S3** Plots of principal component analysis of salivary and dental microbiota based on unweighted and weighted UniFrac metrics. Each point represents the microbiota composition of one participant. (**a**) Unweighted PCoA of dental samples. (**b**) Unweighted PCoA of salivary samples. (**c**) Weighted PCoA of dental samples. (**d**) Weighted PCoA of salivary samples.

**Figure S4** LEfSe identifies the phylotypes with greatest abundance differences between ASD patients and healthy controls for salivary samples (**a**). (Red) phylotypes statistically overrepresented in ASD salivary samples; (green) phylotypes overrepresented in control salivary samples. LEfSe analysis was also performed for dental samples (**b**). (Red) phylotypes statistically overrepresented in ASD dental samples; (green) phylotypes overrepresented in control dental samples. Each filled circle represents one phylotype. The brightness of each dot is proportional to the effect size.

**Figure S5** Diagnostic value of dental samples. (**a**) The AUCs of all combinations in dental plaque samples are depicted in the upper part. The following part shows the magnified portion in the grey rectangle, indicating the AUC of the MIA_AH0HS6 combination reached the highest value. (**b**) ROC curve of the above MIA in ASD diagnosis was depicted. The area under the ROC curve (AUC) was calculated and shown in the center. The arrow indicates the optimal cut-off point in the salivary microbiota.
